# Clinical outcomes and glycaemic responses to different aerobic exercise training intensities in type II diabetes: a systematic review and meta-analysis

**DOI:** 10.1186/s12933-017-0518-6

**Published:** 2017-03-14

**Authors:** Aimee Grace, Erick Chan, Francesco Giallauria, Petra L. Graham, Neil A. Smart

**Affiliations:** 1 School of Science and Technology, University of New England, Armidale, NSW 2351 Australia; 2 School of Medicine, Griffith University, Gold Coast, Australia; 3 Division of Internal Medicine and Cardiac Rehabilitation, Department of Translational Medical Sciences, Federico II University of Naples, Naples, Italy; 4 Department of Statistics, Macquarie University, North Ryde, Sydney, NSW 2113 Australia

**Keywords:** Exercise intensity, Cardio-respiratory fitness, Type II diabetes

## Abstract

**Aims:**

To establish if aerobic exercise training is associated with beneficial effects on clinical outcomes and glycaemic profile in people with type II diabetes.

**Methods:**

A systematic search was conducted to identify studies through a search of MEDLINE (1985 to Sept 1, 2016, Cochrane Controlled Trials Registry (1966 to Sept 1, 2016), CINAHL, SPORTDiscus and Science Citation Index. The search strategy included a mix of MeSH and free text terms for related key concepts. Searches were limited to prospective randomized or controlled trials of aerobic exercise training in humans with type II diabetes, aged >18 years, lasting >2 weeks.

**Results:**

Our analysis included 27 studies (38 intervention groups) totalling 1372 participants, 737 exercise and 635 from control groups. The studies contain data from 39,435 patient-hours of exercise training. Our analyses showed improvements with exercise in glycosylated haemoglobin (HbA1C%) MD: −0.71%, 95% CI −1.11, −0.31; p value = 0.0005. There were significant moderator effects; for every additional week of exercise HbA1C% reduces between 0.009 and 0.04%, p = 0.002. For those exercising at vigorous intensity peak oxygen consumption (peak VO_2_) increased a further 0.64 and 5.98 ml/kg/min compared to those doing low or moderate intensity activity. Homeostatic model assessment of insulin resistance (HOMA-IR) was also improved with exercise MD: −1.02, 95% CI −1.77, −0.28; p value = 0.007; as was fasting serum glucose MD: −12.53 mmol/l, 95% CI −18.94, −6.23; p value <0.0001; and serum MD: −10.39 IU, 95% CI −17.25, −3.53; p value = 0.003.

**Conclusions:**

Our analysis support existing guidelines that for those who can tolerate it, exercise at higher intensity may offer superior fitness benefits and longer program duration will optimize reductions in HbA1C%.

**Electronic supplementary material:**

The online version of this article (doi:10.1186/s12933-017-0518-6) contains supplementary material, which is available to authorized users.

## Background

Meta-analyses have shown lifestyle (diet and exercise) interventions to be beneficial for managing type II diabetes [1]. Furthermore it has been demonstrated that different exercise training modalities produce different effects on glycaemic control in those with type II diabetes, with combined aerobic and resistance exercise reported to be most beneficial [2, 3]. The gold standard measurement of fitness peak oxygen consumption (peak VO_2_) may also be improved [4] as well as glycaemic control [5] in individuals with type II diabetes. In the general population, high intensity interval training has shown to be more effective in regulating glucose than continuous training at lower intensity [6]. Moreover high intensity exercise training has been shown to be superior to lower intensity exercise for improving peak VO_2_ in cardiac patients [7, 8].

Higher-volume high intensity training (HIIT) with 4 × 4 min per session elicited greater improvements than 1 × 4 min of HIIT, or moderate intensity training, in insulin quality in metabolic syndrome (MetS) participants without type 2 diabetes. Both home-based and hospital-based HIIT in cardiac rehabilitation induce promising long-term exercise adherence [9]. However, there are reasons why moderate intensity exercise training is preferred to high intensity, even though health benefits may be greater with the latter. These reasons are: the stimulus from exercise at any intensity is considered sufficient to exert clinical improvements; intuitively one may consider lower intensity exercise training mitigates the likelihood of exercise related medical events; low to moderate intensity exercise can be performed by most people [7].

The life of a red blood cell is approximately 4 months, but many exercise training studies are of shorter duration than this. Glycosylated haemoglobin (HbA1C%) is the blood marker that quantifies the 3-month average plasma glucose concentration. Intuitively an exercise program would take longer than 12 weeks to demonstrate an effect on glycosylated haemoglobin HbA1C%, however many studies are of 12 weeks or fewer duration. It is therefore, also of interest to examine the effect of exercise training duration on HbA1C% and other markers of glycaemic control.

We conducted a systematic analysis of all clinical randomized, controlled, aerobic exercise training trials in people with type II diabetes and stratified the trials by exercise intensity according to recognized guidelines [10]. Our systematic review and meta-analysis work had three aims: First, we aimed to quantify the effect of aerobic exercise on change in markers of glycaemic control and peak VO_2_ compared to sedentary controls. Second, we aimed to establish if high/vigorous intensity interval training produces larger changes in markers of glycaemic control and peak VO_2_ compared to moderate/low intensity training and sedentary controls. Third, we wished to establish if exercise training duration produces better glycaemic control and peak VO_2_.

## Methods

### Search strategy

Studies were identified through a MEDLINE search strategy (1985 to Sept 1, 2016), Cochrane Controlled Trials Registry (1966 to Sept 1, 2016), CINAHL, SPORTDiscus and Science Citation Index. The search strategy included a mix of MeSH and free text terms for the key concepts related to exercise training, high intensity interval exercise, peak VO_2_, type II diabetes, glycaemic control, insulin sensitivity and insulin resistance for clinical trials of exercise training in people with type II diabetes. Studies were included if patients exhibited a diagnosis of type II diabetes. Searches were limited to prospective randomized, controlled trials of aerobic exercise training in humans, aged >18 years, lasting 2 weeks or more, supervised and unsupervised program were included. No restrictions were placed on the year, or language, of publication. Reference lists of papers and latest editions of relevant journals which were not available online were scrutinised for new references. Full articles were read and assessed by three reviewers (NS, AG and EC) for relevance and study eligibility. Disagreements on methodology were resolved by discussion, a fourth reviewer (FG) adjudicated over any disputes. Study authors were contacted and requested to provide further data if required.

### Study selection

Included studies were randomized controlled trials of exercise training, of 6 weeks or longer, supervised or unsupervised, in people with type II diabetes. Although some outcomes such as HbA1C% are unlikely to change in less than 12 weeks, peak VO_2_ etc. will change in this time period [11, 12]. Studies of type I diabetes were excluded to maintain a homogeneous study population and the type I patients may have made up disproportionately fewer total patients. Resistance training studies were excluded as they tend not to use or measure peak VO_2_ as an outcome measure and there are too few isolated resistance studies to warrant an analysis. As a result resistance study data were not included in our analysis. All published studies included in this systematic review were comparisons between exercise study groups and control. Reviewers categorized the studies into four groups based on exercise intensity. The categorization were based on the position stand by Exercise and Sport Science Australia [10]. The measures used to classify exercise intensity were percentage of Heart Rate Maximum (%HR_max_), Heart Rate Reserve (%HRR), Peak Oxygen Uptake (%VO_2_ Peak) and Borg scale score [10].

In addition to the records identified through database searching, reference lists of identified records were scrutinized. Only the principal study with the greatest number of subjects were included where multiple publications existed from the same dataset. After initial screening over-lapping, duplicates, duplicate data and irrelevant articles such as editorials and discussion papers that did not match the inclusion criteria were removed. We excluded studies where the control group received additional intervention, non-relevant studies; studies using non-aerobic exercise training and those reporting only acute exercise testing responses. We excluded studies from specific analyses if incomplete data was reported and the authors did not respond to our requests to provide missing data.

### Outcomes measures

We recorded the following data; percentage change in HbA1C%, Homeostatic model assessment of insulin resistance (HOMA-IR), lean body mass, BMI, body composition, peak VO_2_ (only where this was measured directly during peak exercise), fasting glucose and insulin at baseline and post exercise. We also recorded exercise training frequency, intensity, duration per-session, length of exercise program.

### Data synthesis

From extracted data we calculated patient-hours of exercise training, percentage change in outcome measures.

### Assessment of study quality

We assessed study quality with regard to: eligibility criteria specified, random allocation of participants, allocation concealed, similarity groups at baseline, assessors blinded, outcome measures assessed in 85% of participants and intention to treat analysis. The study quality was assessed according to the validated Tool for the assEssment of Study qualiTy and reporting in EXercise (TESTEX). TESTEX is a study quality and reporting assessment tool, designed specifically for use in exercise training studies. which has a maximum score of 15 [13]. The main point of difference in TESTEX is that there are accommodations for: Activity monitoring in control groups to measure crossover to exercise by sedentary control patients; Assessment of the existence and method of activity monitoring in both exercise intervention and sedentary controls; Assessment of whether the relative exercise intensity remained constant and therefore potentially avoided de-training as participants initially adapt to new exercise programs; Assessment of whether periodic evidence-based adjustment of exercise intensity is reported exercise volume and exercise expenditure Information on all exercise characteristics (intensity, duration, frequency and mode) is provided to calculate exercise volume and exercise energy expenditure.

This tool is a 15-point scale (5 points for study quality and 10 points for reporting) and addresses previously unmentioned quality assessment criteria specific to exercise training studies. Two reviewers NS and FG conducted the risk of bias assessment, PG was consulted of discrepancies occurred. No minimum TESTEX score was required for a study to be included in the analysis.

### Statistical analyses

A mixed-effects meta-analysis model was used to estimate the effect of exercise versus control for the outcomes of interest while controlling for the repeated measures arising in multi-arm studies and over time for the same study. The *metafor* [14] package within R statistical software was used to conduct the meta-analyses and create forest plots [15].

Continuous outcomes were reported as mean difference between exercise and control and/or relative change from baseline scores depending on the data extracted with 95% confidence intervals (CI).

We used a 5% level of significance to report differences between intensity and control in each of the outcome measures. Egger bias tests were employed if at least 10 studies were included for a given outcome [16]. Heterogeneity is presented as the estimated between studies variability, τ^2^ with 95% CI. A multivariate generalization of the I^2^ statistic is also provided.

## Meta-regression

Meta-regression was performed to determine whether exercise volume variables and possible predictors (exercise intensity, program duration and patient-hours of exercise, publication date) of change in outcome measures mediated the differences between treatment and control. This was not undertaken if there were fewer than 10 studies in a given analysis.

## Results

**Fig. 1:**
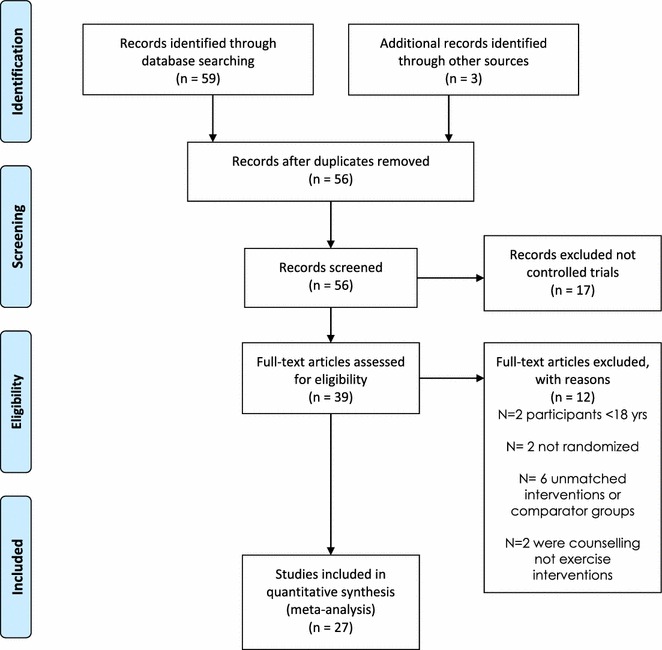
Consort statement

Our initial search identified 59 manuscripts, hand searching of reference lists of included studies and key articles such as related reviews and the latest editions of relevant journals yielded a further 3 manuscripts. Out of these 62 studies, 6 were excluded at first inspection as duplicates, 17 were not controlled trials of exercise therapy, 2 were excluded as they had participants <18 years, 2 were excluded as they were not randomized trials, 6 used unmatched interventions or comparator groups and 2 were counselling interventions encouraging exercise participation, leaving 27 included studies for analysis (PRISMA Statement—Fig. 1).

The 27 studies (38 intervention groups) comprised 1372 participants, 737 exercise and 635 from control groups [3, 17–42]. The studies contain data from 39,435 patient-hours of exercise training. Studies ranged in duration form 4–52 weeks (average 17.8 weeks, median 13 weeks), and 2–5 weekly exercise sessions (median = 3), session duration ranged from 15 to 75 min (median = 50), mean weekly exercise time was 40–300 min (mean 157 min, median = 150 min). All were studies of aerobic exercise, 24 intervention groups used vigorous intensity, 1 used low intensity, 1 was unclassified with regards to intensity and 12 used moderate intensity (see Table 1).

**Table 1:** Included study characteristics

Study	Date	Country	No. exercise	No. control	Weeks	Exercise intensity
Agurs-Collins et al. 12 weeks	1997	USA	32	32	12	Moderate
Agurs-Collins et al. 24 weeks	1997	USA	32	32	24	Moderate
Balducci et al. 13 weeks	2010	Italy	20	20	13	Vigorous
Balducci et al. 26 weeks	2010	Italy	20	20	26	Vigorous
Balducci et al. 39 weeks	2010	Italy	20	20	39	Vigorous
Balducci et al. 52 weeks	2010	Italy	20	20	52	Vigorous
Belli et al.	2011	Brazil	9	10	12	Moderate
Boudou et al.	2000	France	8	8	9	Vigorous
Choi et al.	2012	Korea	38	37	12	Moderate
Church et al.	2010	USA	72	41	39	Vigorous
Cuff et al.	2003	Canada	9	9	16	Vigorous
de Oliveira	2012	Brazil	11	12	12	Moderate
da Silva et al. low	2011	Brazil	10	11	6	Low
da Silva et al. vigorous	2012	Brazil	10	11	6	Vigorous
Jorge	2011	Brazil	12	12	12	Moderate
Kadoglou	2007	Greece	30	30	26	Vigorous
Karstoft et al. continous	2013	Denmark	12	8	17	Moderate
Karstoft et al. interval	2013	Denmark	12	8	17	Vigorous
Lambers	2008	Belgium	19	16	12	Vigorous
Madden	2013	Canada	10	10	12	Vigorous
Mitranun et al. continuous	2014	Thailand	14	15	12	Moderate
Mitranun et al. interval	2014	Thailand	14	15	12	Vigorous
Moghadasi	2013	Iran	8	8	12	Moderate
Morton	2010	UK	15	12	7	Vigorous
Motahari-Tabari et al. 4 weeks	2014	Iran	27	26	4	Moderate
Motahari-Tabari et al. 8 weeks	2014	Iran	27	26	8	Moderate
O’Donovan et al. moderate	2005	UK	10	13	24	Moderate
O’Donovan et al. vigorous	2005	UK	13	13	24	Vigorous
Raz et al.	1994	Israel	19	19	12	Vigorous
Rönnemaa	1986	Finland	15	15	17	Vigorous
Shenoy	2010	India	20	20	8	Vigorous
Short	2003	USA	65	37	16	Vigorous
Sigal 13 weeks	2007	Canada	60	63	13	Vigorous
Sigal 26 weeks	2007	Canada	60	63	26	Vigorous
Sridhar	2010	India	30	22	52	Unclear
Sung 13 weeks	2012	Korea	22	18	13	Vigorous
Sung 26 weeks	2012	Korea	22	18	26	Vigorous
Yavari	2012	Iran	35	30	16	Vigorous

## Meta-analyses

### HbA1C%

**Fig. 2:**
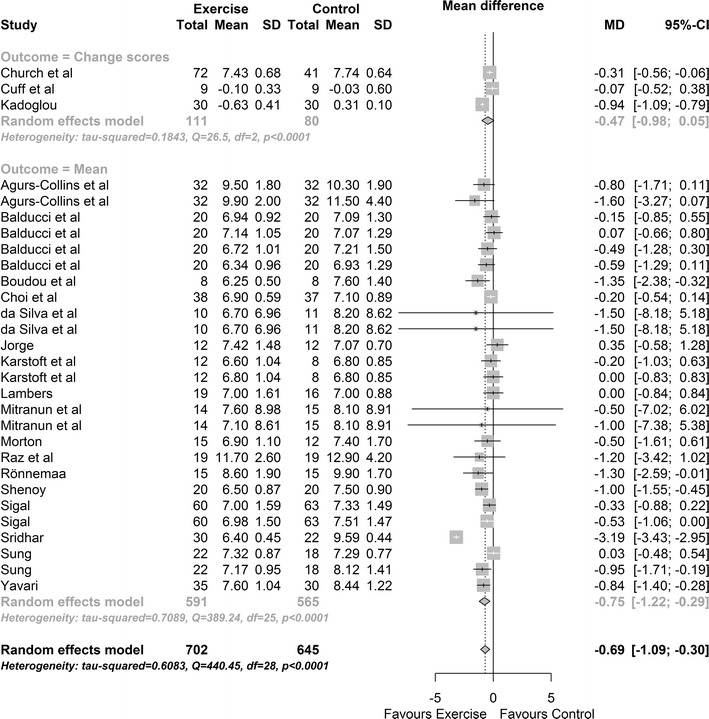
Change in HbA1C% exercise vs control

Twenty intervention groups provided data on HbA1C%. Results indicated that there was a significant reduction in the exercise versus control groups HbA1C%, MD: −0.69%, 95% CI −1.09, −0.30; p value = 0.0005 (Fig. 2). Cochran’s Q-test indicates significant heterogeneity between studies (Q = 440, df = 28, p value <0.0001; between studies variability: τ^2^ = 0.61, 95% CI 0.31, 1.36; I^2^ = 89.8%). An Egger bias test indicated no evidence of funnel plot asymmetry (p value = 0.710). There was one significant moderator effect; exercise program duration (weeks). For every additional week of follow-up HbA1C% reduces between 0.009 and 0.043%, p = 0.002.

### HOMA-IR

**Fig. 3:**
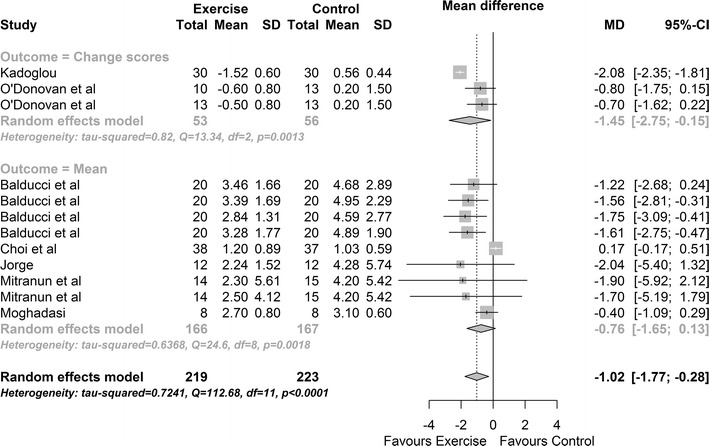
Change in HOMA-IR exercise vs control

Seven studies provided data on HOMA-IR. Results indicated a significant improvement in HOMA-IR in exercise participants versus control MD: −1.02, 95% CI −1.77, −0.28; p value = 0.007 (Fig. 3). Cochran’s Q-test indicated significant heterogeneity between studies (Q = 113, df = 11, p value <0.0001; between studies variability: τ^2^ = 0.72, 95% CI 0.22, 3.54; I^2^ = 83.7%). There were too few studies to allow investigation of moderator effects or to perform an Egger bias test.

### Fasting insulin

Eight intervention groups provided data on insulin. Results suggested a significant reduction in serum insulin in the exercise participants versus control MD: −10.39 IU, 95% CI −17.25, −3.53; p value = 0.003 (see Additional file 1). Cochran’s Q-test indicates significant heterogeneity between studies (Q = 304, df = 10, p value <0.0001; between studies variability: τ^2^ = 136.8, 95% CI 41.0, 646.0; I^2^ = 94.5%). There were too few studies to allow investigation of moderator effects or to perform an Egger bias test.

### Fasting blood glucose

Eighteen studies provide data on fasting blood glucose. Results indicated that there was a significant reduction in serum glucose in exercise participants versus control MD: −12.53 g.DL^−1^, 95% CI −18.94, −6.23; p value <0.0001 (see Additional file 1). Cochran’s Q-test indicates significant heterogeneity between studies (Q = 522, df = 23, p value <0.0001; between studies variability: τ^2^ = 136.1, 95% CI 56.8, 361.9; I^2^ = 94.2%). An Egger bias test indicated no evidence of funnel plot asymmetry (p value = 0.862). There were no significant moderator effects.

### Body mass index

Nineteen intervention groups were analysed for body mass index (BMI) which suggested significantly reduced BMI in exercise versus control participants MD—1.56 kg m^−2,^ 95% CI −2.41, −0.71; p value = 0.0003 (see Additional file 1). Cochran’s Q-test showed no evidence of heterogeneity between studies (Q = 27, df = 26, p value = 0.39; between studies variability: τ^2^ = 0.78. 95% CI 0.00; 4.71; I^2^ = 36.4%). An Egger bias test showed no evidence of funnel plot asymmetry (p value = 0.884). There were no significant moderator effects.

### Lean body mass

Six studies were included in the analysis. Results for lean body mass indicated no difference between exercise and control groups MD: −0.44 kg 95% CI −1.19, 0.31; p value = 0.246 (see Additional file 1). Cochran’s Q-test indicates no evidence of heterogeneity between studies (Q = 1.28, df = 7, p value = 0.991; between study variability: τ^2^ = 0.00, 95% CI 0.00, 8.00; I^2^ = 0.00%). There were too few studies to test for moderator effects or funnel plot asymmetry.

### Fat mass

Six intervention groups provided data on fat mass. Results indicate that there was no difference between exercise and control MD: −0.47 kg 95% CI −1.54, 0.61; p value = 0.396 (see Additional file 1). Cochran’s Q-test indicates no evidence of heterogeneity between studies (Q = 5, df = 7, p value = 0.703; between studies variability: τ^2^ = 0.00, 95% CI 0.00, 10.0; I^2^ = 0.00%). There were too few studies to be able to test for moderator effects or funnel plot asymmetry.

### Cardiorespiratory fitness (peak VO_2_)

**Fig. 4:**
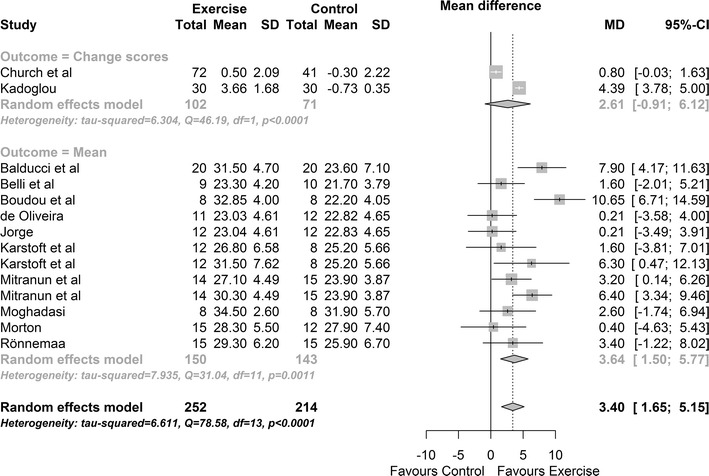
Change in peak VO_2_ exercise vs control

Twelve intervention groups provided data on peak VO_2_ (ml/kg/min). Results show a significant improvement in exercise participants versus control MD: 3.40 ml/kg/min, 95% CI 1.65, 5.15; p value = 0.0001 (see Fig. 4). Cochran’s Q-test indicates significant heterogeneity between studies (Q = 79, df = 13, p value <0.0001; between studies variability: τ^2^ = 6.61, 95% CI 1.72, 23.2; I^2^ = 85.3%). There was no evidence of funnel plot asymmetry (Egger bias test p value = 0.815). There was a significant moderator effect; participants undertaking vigorous exercise had significantly higher peak VO_2_ max (between 0.64 and 5.98 ml/kg/min more) than participants undertaking low/moderate exercise (p = 0.015).

### Study quality

Median TESTEX score was 7. Several aspects of study design were conducted poorly on more than 50% (13) studies; the method of randomization was only clearly stated in 9/27 studies; group allocation was only concealed from assessors in 5 studies; assessor blinding was only employed in 4 studies; intention to treat analysis was only done in 3 studies; physical activity monitoring of controls was only performed in 1 study; exercise intensity was periodically reviewed in only 5 studies (see Additional file 1: Table S2).

## Discussion

Our work is the first to conduct a data pooling analysis of the effects of exercise training and associated moderator variables on clinical markers of diabetes control. Our analyses showed improvements in HbA1C%, HOMA-IR, serum insulin and glucose and peak VO_2_. There remains insufficient published data to conduct moderator effects in but a handful of the reported outcome measures, however it is likely that exercise program duration and intensity have a moderating role.

HbA1C% showed a significant improvement with exercise training. As the median duration of included studies was 13 weeks, which is a similar duration to the life of a red blood cell, it is therefore encouraging that changes in HbA1C% via exercise training are possible in such a short timeframe. We conducted sub-analyses and found that for every extra week of exercise training one can expect a reduction in HbA1C% compared to control. While this is effect is small it reinforces the importance of sustained lifestyle changes to effect health improvements.

Our work showed that exercise training improved HOMA-IR. HOMA-IR values above or equal to 2.0 or 2.5 show enhanced diagnostic value in distinguishing those with metabolic disease from healthy individuals [43]. Our work showed exercise training elicited a mean difference improvement in HOMA-IR of greater than 1.0 indicating a large effect size. One would expect that a reduction in HOMA-IR of 1.0 would have a profound effect on the metabolic profile of an individual. We were unable to establish if any of the moderator variables influenced the magnitude of change in HOMA-IR.

We found BMI to be favourably altered with exercise. While point estimates showed a reduction in lean body mass and fat body mass the reductions were not significant in exercising participants versus sedentary controls though this may be due to the small number of studies combined. No moderator variables were found to influence the magnitude of these changes. Our findings are expected in participants who exhibit improvements in HbA1C% and HOMA-IR.

The change in Peak VO_2_ was the order of 1 MET and this moderate effect is to be expected from a known chronic disease group who notoriously exhibit sedentary behaviour. Our meta-regression analysis of exercise intensity found a more pronounced increase in peak VO_2_ with vigorous versus low-moderate intensity training. Previous work has shown that intensity is the primary stimulus for improved peak VO_2_ in people with cardiac disease [8, 44]. It is remarkable that the exercise recommendations for diabetes were one of the first to offer a sliding exercise prescription scale, based upon the manipulation of intensity and weekly duration in order to keep work volume relatively constant [45]. These guidelines suggest 270 weekly minutes of moderate intensity exercise but only 90 min of vigorous intensity activity. Our work supports the existence of a two-tiered exercise prescription as exercise at vigorous intensity is likely to produce a small to moderate reduction, beyond that observed with moderate intensity exercise, in HbA1C%.

We did not observe any moderating effects of exercise program duration on peak VO_2_. One may expect intuitively that increasing exercise program duration would produce greater improvements in peak VO_2_. It is therefore perhaps surprising that studies comparing shorter and longer exercise program durations have produced non-uniform effects on peak VO_2_ [7, 8, 46]. The likely explanation for this phenomenon is that it may be more difficult to get patients to continue to adhere to an exercise program in the longer term.

There is a broad consensus that physical activity represents a natural strong anti-inflammatory and metabolism-improving strategy with minor side effects, this is likely to be true in people with T2DM undertaking aerobic training, even in water can to reduce glucose levels in this patient group [47, 48]. Moreover, there is a quantitative relationship between HbA1c levels and plaque texture in ultrasonic images of atherosclerotic patients [49], and in those without identified carotid plaques, beneficial effect of exercise training on carotid IMT progression has been demonstrated [50]. On the other hand, these favorable effects have not been always seen [51].

### Study limitations

Most of the meta-analyses exhibited heterogeneity that was not substantially reduced through a systematic attempt to identify reasons for heterogeneity by grouping studies according to similarities in outcome reporting or meta-regression.

The exercise training programs varied greatly between studies with respect to exercise intensity, duration, frequency and modality. We accounted for duration via the statistical model used and the other variables via meta-regression however, for the most part the meta-regressions were not significant. Despite the heterogeneous study designs, the Egger bias tests suggested no evidence of funnel plot asymmetry suggesting minimal risk of publication bias.

Few included studies accurately quantified the volume of incidental and structured physical activity, this has been performed previously in people with T1DM [52].

Measures of lean, and fat, body mass would have shed more light onto the role that body composition plays in improving glycaemic control through exercise. We would like to have conducted more moderator variable analyses but limited extracted data precluded this. We were only able to consider program duration, and high/vigorous versus low/moderate exercise intensity sub-analyses.

Finally, whether comorbidity (i.e. concomitant cardiac disease, chronic obstructive pulmonary disease, etc.) impact on exercise-induced improvement of peak VO_2_ and glycaemic profile according to exercise training modalities remains to be elucidated.

## Conclusions

Our pooled analysis of aerobic exercise studies showed a significant improvement in both HbA1C% and peak VO_2_. Moreover our data support existing guidelines that for those who can tolerate it, exercise at higher intensity may offer superior benefits. Longer exercise program duration will also optimize reductions in HbA1C%.

## Additional file

**Additional file 1.** Additional figures and table.

## Abbreviations

HbA1C%: glycosylated haemoglobin
Peak VO_2_: peak oxygen consumption
HOMA: IR homeostatic model assessment of insulin resistance
MetS: metabolic syndrome
HIIT: High Intensity Intermittent Training
%HR_max_: heart rate maximum
%HRR: percentage of heart rate reserve
%VO_2_ Peak: percentage of peak oxygen uptake
MD: mean difference
