# Supplementary material for: Clinical outcomes and glycaemic responses to different aerobic exercise training intensities in type II diabetes: a systematic review and meta-analysis
Source: Cardiovasc Diabetol. 2017 Mar 14;16:37. doi: 10.1186/s12933-017-0518-6 (PMC5351065; doi:10.1186/s12933-017-0518-6)
Supplement: Supplementary file 1 — Additional file 1. Additional figures and table. [file 12933_2017_518_MOESM1_ESM.docx]

**Supplementary Files**

Figure S1. Change in Insulin


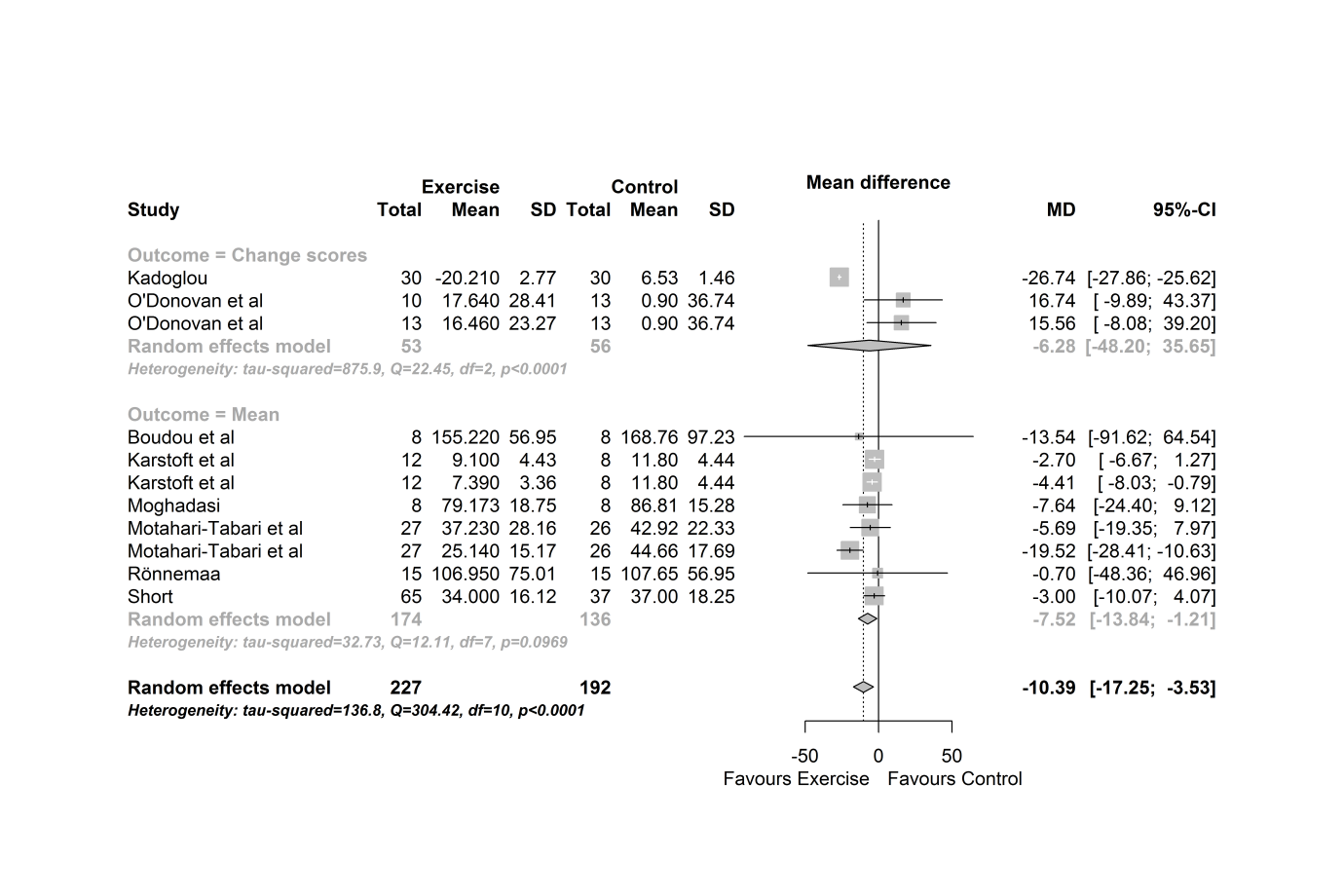


Figure S2 Change in Blood Glucose


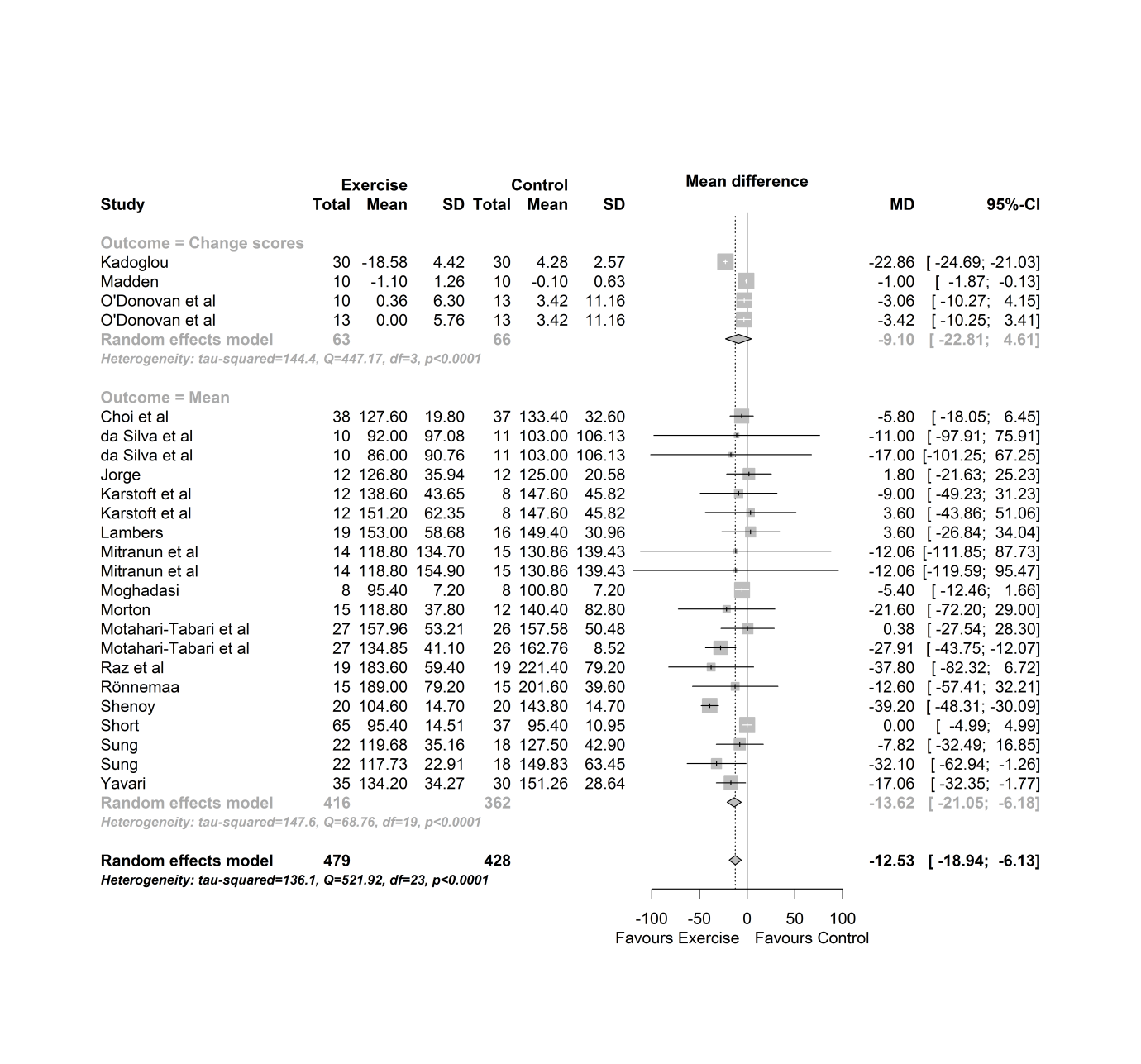


**Figure S3 Change in Body Mass Index**

**
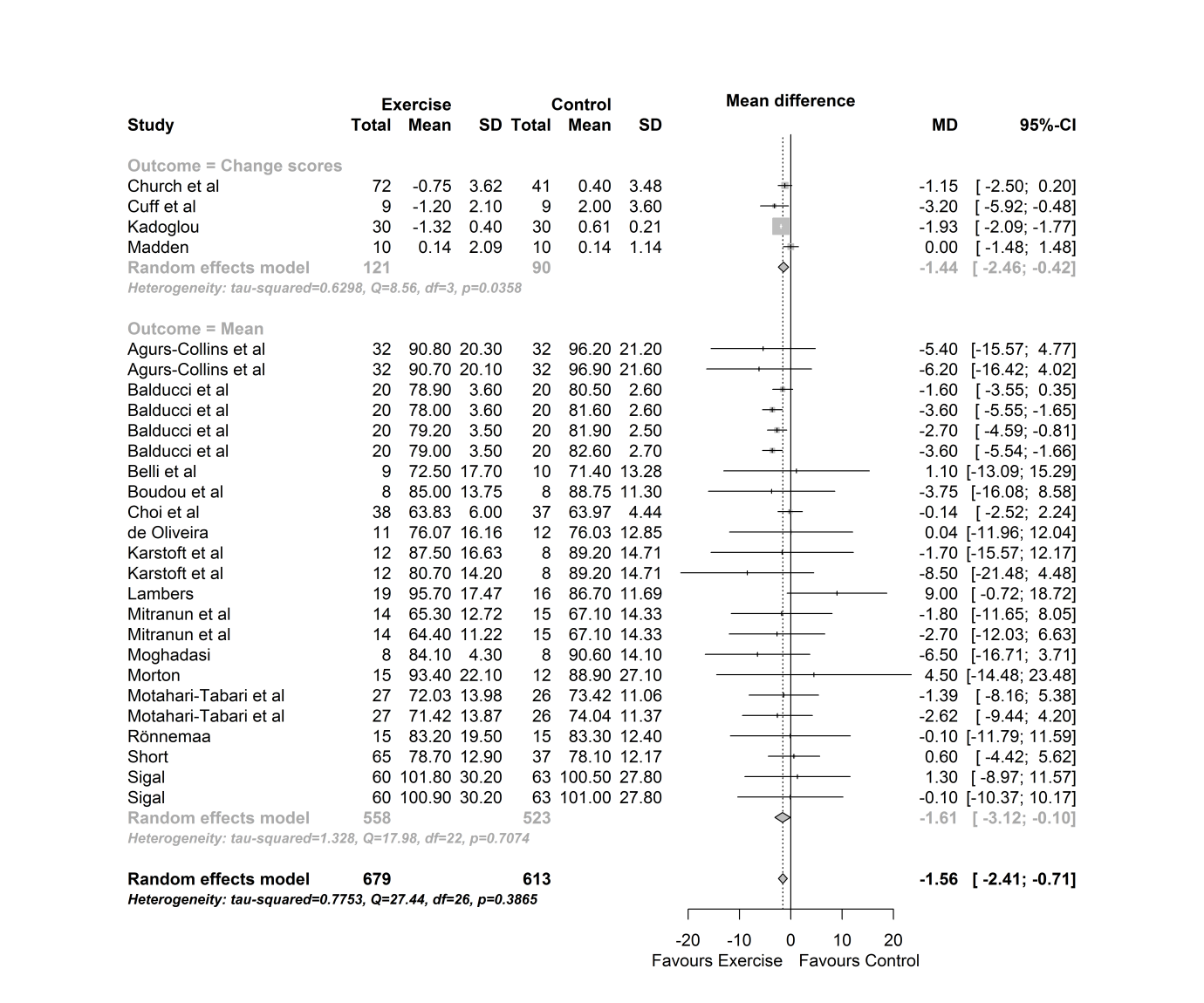
**

**Figure S4 Change in Lean Body Mass**

**
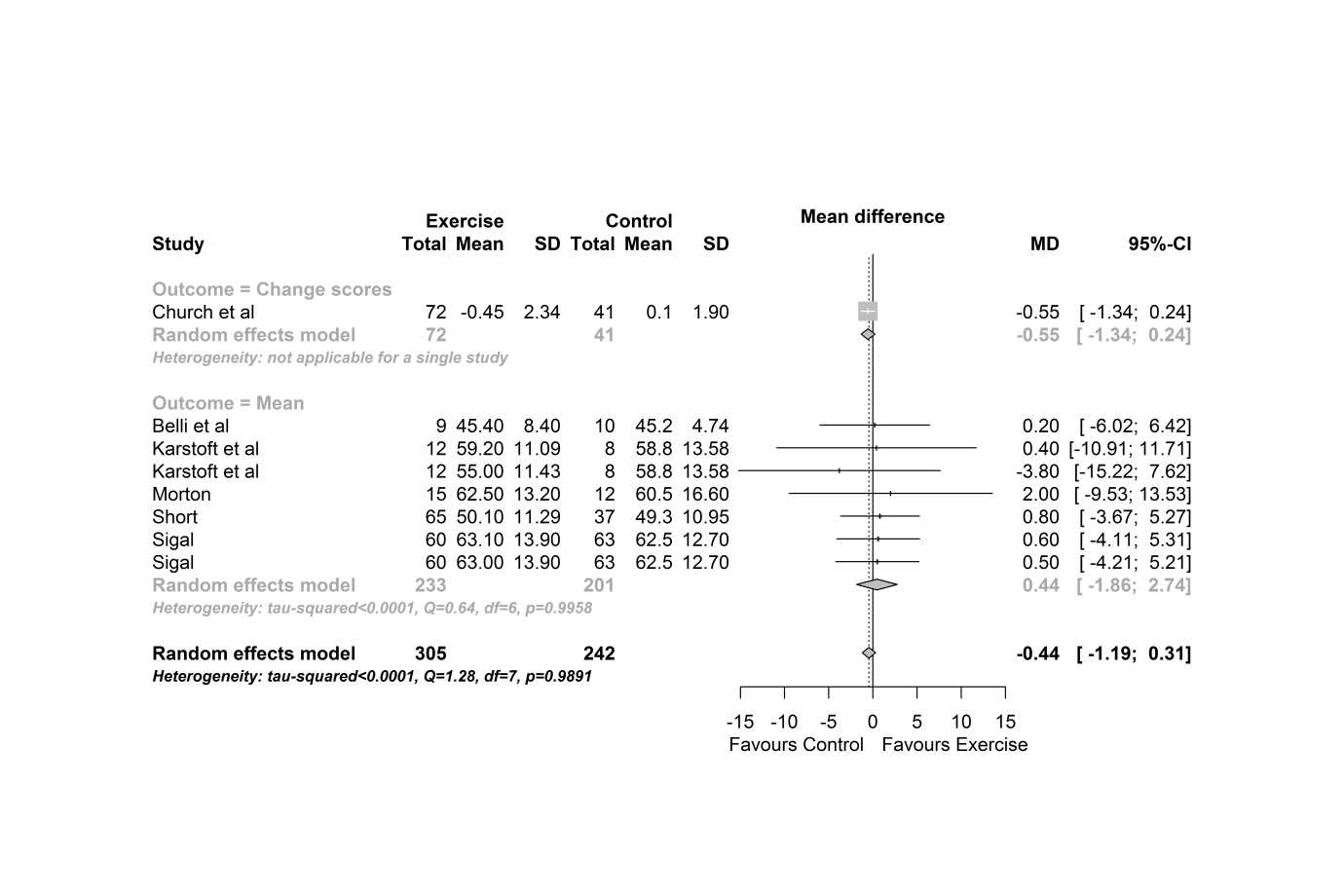
**

**Figure S5 Change in Fat Mass**

**
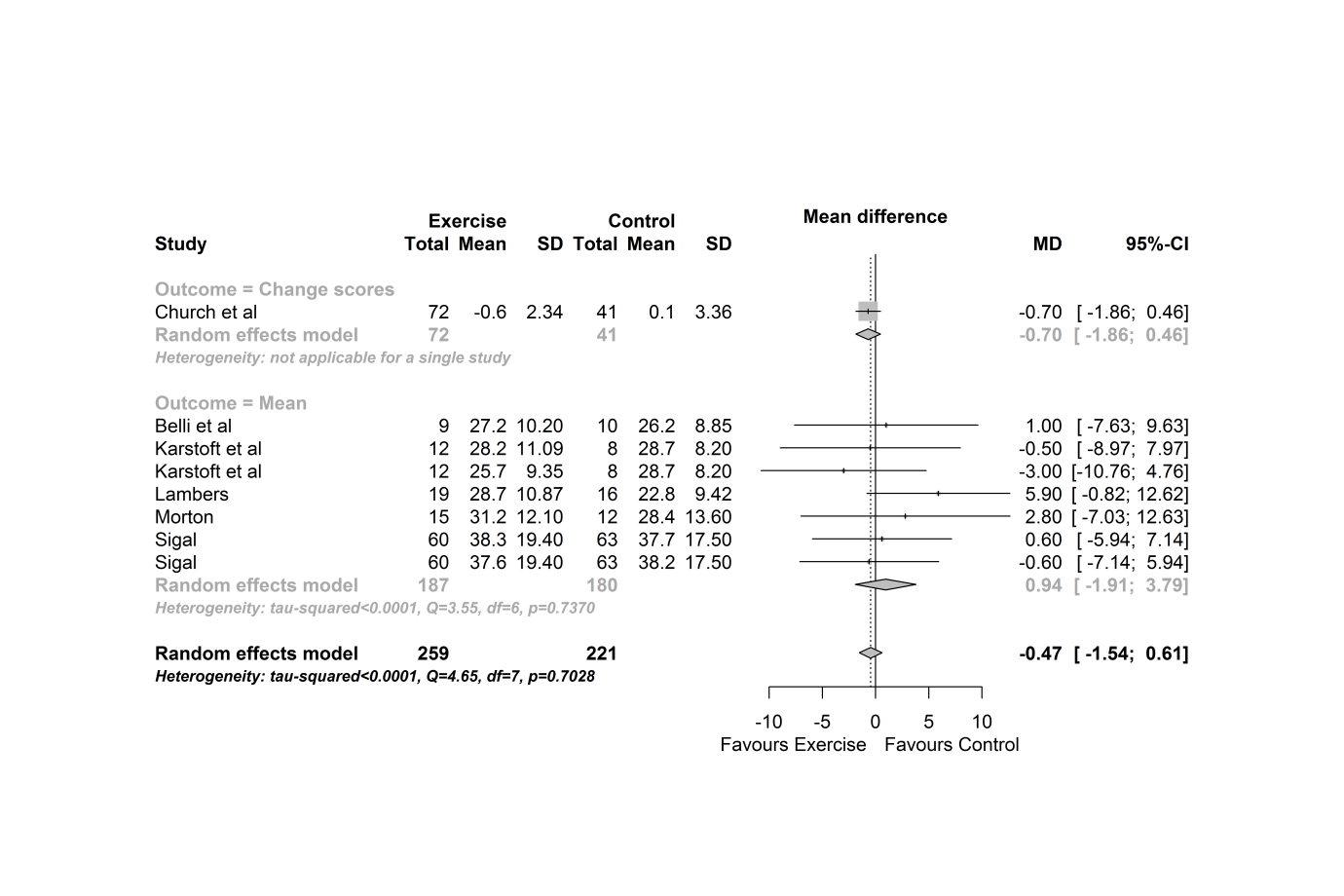
**

**Figure S6 Change in Lean Body Mass Percentage**

**
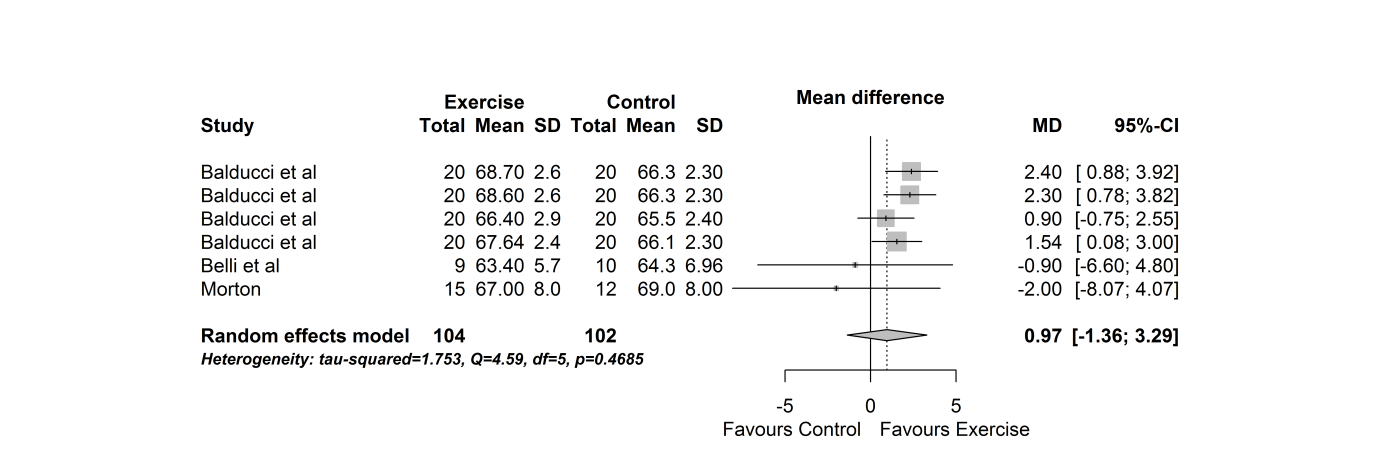
**

**Table S2. Study Quality Assessment of Included Studies using the Tool for the Assessment of Study Quality in Exercise Training (TESTEX)**

| Study name | Eligibility  Criteria  specified | Randomly  allocated  participants | Allocation  concealed | Groups  Similar at  baseline | Assessors  blinded | | Outcome  Measures  assessed >85%  of participants# | | Intention  to treat  analysis | Reporting of  between group  statistical  comparisons | | Point measures  & measures  of variability  reported* | Activity  Monitoring  in Control  Group | Relative  Exercise  Intensity  Review | Exercise  Volume &  Energy  Expended | Overall TESTEX |
| --- | --- | --- | --- | --- | --- | --- | --- | --- | --- | --- | --- | --- | --- | --- | --- | --- |
| AGURS-COLLINS (1997) | YES | YES | YES | YES | NO | | YES (2) | YES | | YES (2) | | YES | NO | NO | NO | 10 |
| BALDUCCI (2010) | YES | NO | NO | YES | YES | | NO | NO | | YES (2) | | YES | NO | NO | YES | 7 |
| BELLI (2011) | YES | YES | NO | YES | NO | | YES (2) | NO | | YES (2) | | YES | NO | NO | YES | 9 |
| BOUDOU (2000) | NO | NO | NO | NO | NO | | YES (1) | NO | | YES (2) | | NO | NO | NO | YES | 4 |
| CHOI (2012) | YES | NO | YES | YES | NO | | NO | NO | | YES (2) | | YES | NO | NO | YES | 7 |
| CHURCH (2010) | YES | YES | NO | YES | YES | | YES (2) | YES | | YES (2) | | YES | NO | NO | YES | 11 |
| CUFF (2003) | YES | NO | NO | YES | NO | | YES (1) | NO | | YES (2) | | YES | NO | NO | YES | 7 |
| DA SILVA (2012) | YES | NO | NO | YES | NO | | YES (1) | NO | | YES (2) | | YES | NO | YES | YES | 8 |
| DE OLIVEIRA (2012) | YES | NO | NO | YES | NO | | YES (1) | NO | | YES (2) | | YES | NO | NO | YES | 7 |
| JORGE (2011) | YES | NO | YES | YES | NO | | YES (1) | NO | | YES (2) | | NO | NO | NO | YES | 7 |
| KADOGLOU (2007) | YES | NO | NO | YES | NO | | YES (3) | NO | | YES (2) | | NO | NO | YES | YES | 9 |
| KARSTOFT (2013) | YES | NO | NO | YES | NO | | YES (1) | NO | | YES (2) | | NO | YES | YES | YES | 8 |
| LAMBERS (2008) | YES | YES | NO | YES | YES | | YES (3) | NO | | YES (2) | | YES | NO | YES | YES | 12 |
| MITRANUN (2014) | YES | NO | NO | NO | NO | | NO | NO | | YES (2) | | YES | NO | YES | YES | 6 |
| MOGHADASI (2013) | YES | NO | NO | YES | NO | | NO | NO | | YES (2) | | YES | NO | NO | NO | 5 |
| MORTON (2010) | YES | NO | NO | YES | NO | | YES (1) | NO | | YES (2) | | YES | NO | NO | YES | 7 |
| MOTAHARI-TABARI (2015) | YES | YES | NO | YES | NO | NO | | NO | | YES (2) | YES | | NO | NO | YES | 7 |
| O'DONOVAN (2005) | YES | YES | YES | YES | NO | NO | | NO | | YES (2) | YES | | NO | NO | YES | 8 |
| RAZ (1994) | NO | NO | YES | YES | NO | YES (2) | | NO | | YES (2) | YES | | NO | NO | YES | 8 |
| RONNEMAA (1986) | NO | NO | NO | YES | NO | NO | | NO | | YES (2) | YES | | NO | NO | YES | 5 |
| SHENOY (2010) | YES | YES | NO | YES | NO | NO | | NO | | NO | YES | | NO | NO | YES | 7 |
| SHORT (2003) | YES | NO | NO | YES | NO | YES (2) | | NO | | YES (2) | YES | | NO | NO | YES | 8 |
| SIGAL (2007) | YES | YES | NO | YES | YES | YES (3) | | YES | | YES (2) | YES | | NO | NO | YES | 12 |
| SUNG (2012) | YES | YES | NO | YES | NO | NO | | NO | | YES (2) | YES | | NO | NO | YES | 7 |
| YAVARI (2012) | YES | NO | NO | YES | NO | YES (1) | | NO | | YES (2) | YES | | NO | NO | YES | 7 |
|  |  |  |  |  |  |  | |  | |  |  | |  |  |  |  |
|  |  |  |  |  |  |  | |  | |  |  | |  |  |  |  |
| Sub-Totals | 24 | 9 | 5 | 25 | 4 | 18 | | 3 | | 26 | 23 | | 1 | 5 | 25 | Median 7 |

Total out of 15 Points

Legend: # Three points possible – 1 point if adherence>85%, 1 point if adverse events reported, 1 point if exercise attendance is reported

*Two points possible – 1 point if primary outcome is reported, 1 point if all other outcomes reported
